# Kinetics of Tear Fluid Proteins after Endothelial Keratoplasty and Predictive Factors for Recovery from Corneal Haze

**DOI:** 10.3390/jcm9010063

**Published:** 2019-12-26

**Authors:** Nobuyo Yawata, Sunita Awate, Yu-Chi Liu, Shi Yuan, Kaing Woon, Jay Siak, Yoh-Ichi Kawano, Koh-Hei Sonoda, Jodhbir S. Mehta, Makoto Yawata

**Affiliations:** 1 Department of Ophthalmology, Kyushu University, Fukuoka 812-8582, Japan; 2 Singapore Eye Research Institute, Singapore 168751, Singapore; 3 Ophthalmology and Visual Sciences Academic Clinical Program, Duke-NUS Medical School, Singapore 169857, Singapore; 4 Singapore Institute for Clinical Sciences (SICS), Agency for Science, Technology and Research, A*STAR, Singapore 117609, Singapore; 5 Singapore National Eye Centre, Singapore 168751, Singapore; 6 Department of Ophthalmology, Yong Loo Lin School of Medicine, National University of Singapore, Singapore 119228, Singapore; 7 Department of Ophthalmology, Fukuoka Dental College, Fukuoka 814-0193, Japan; 8 Department of Pediatrics, Yong Loo Lin School of Medicine, National University of Singapore, Singapore 119228, Singapore; 9 National University Health System, Singapore 119228, Singapore; 10 Immunology Programme, Life Sciences Institute, National University of Singapore, Singapore 117456, Singapore; 11 International Research Center for Medical Sciences, Kumamoto University, Kumamoto 860-8555, Japan

**Keywords:** endothelial keratoplasty, tear fluid cytokines, corneal haze

## Abstract

Endothelial keratoplasty (EK) is less invasive with faster recovery as compared to conventional penetrating keratoplasty, however, it relies on the clarity of the host corneal stroma. Corneal transplantation involves the induction of immune tolerance for allogeneic tissues as well as the corneal wound healing process, in which coordinated interactions between cytokines and growth factors are critical. In this study, we profiled the expression of 51 soluble factors in the tear fluid over the course of EK and have provided evidence of dynamic changes in cytokine expression in the ipsilateral and contralateral eyes. Cluster analyses classified the cytokine expression kinetics into five groups. Group 1 proteins included TGF-b1, IL-1b, and innate proinflammatory cytokines, which bilaterally increased after surgery, despite the use of topical corticosteroid in the transplanted eyes. Local corticosteroids suppressed cytokines involved in adaptive immunity in the transplanted eyes but not in the contralateral eyes. We found tear protein expression at baseline and one week post-surgery to be a potential predictive biomarker of delayed recovery after EK in terms of the corneal haze and visual acuity. Furthermore, Group 1 tear proteins were most associated with persistent corneal haze pre-surgery as well as visual acuity at one month-post transplant.

## 1. Introduction

Corneal transplantation is evolving from total replacement of the cornea tissue layers (epithelium, stroma and endothelium: Penetrating Keratoplasty; PKP), toward selective tissue stripping of the endothelium and subsequent allogeneic transplantation of the endothelium transplantation: Descemet’s stripping automated endothelial keratoplasty (DSAEK) and Descemet’s membrane endothelial keratoplasty (DMEK). These newer techniques have come to be preferred because they are less invasive, and have less complications and faster recovery times [1,2]. There is, however, substantial variation in recovery rates amongst the transplant cases.

Issues in corneal transplant commonly encountered are delayed recovery of corneal transparency (“haze”), graft rejection, and decompensation (functional failure of the transplanted endothelium). Successful outcomes in corneal transplantation require synchronized expression amongst cytokines, chemokines, and growth factors produced by corneal epithelial, stromal, endothelial cells, and inflammatory cells. The current perspective is that an orderly progression in the cross-talk between these various factors is important in determining transplant outcomes [3,4,5,6,7].

In the corneal tissue, a tissue repair process is thought to initiate immediately following transplantation. The TGF-b and IL-1 families are master regulators of corneal wound healing [8,9]. TGF-b1/b2 are profibrotic cytokines, which play a critical role in the activation of corneal keratocytes to myofibroblasts, enabling the tissue repair. In the latter stages of wound healing, a key to successful cornea repair is the timely clearance of these myofibroblasts. IL-1 induces the apoptosis of activated myofibroblasts, and initiates an inflammatory response by upregulating IL-6, IL-8, and MCP-1 [10,11,12]. Under an ideal post-transplant process, reduction in corneal haze is cleared rapidly. The change in the degree of haze is thus an objective clinical feature that enables assessment of surgical wound healing. The factors that contribute to haze are underexplored, and likely involve responses by the tissue microenvironment and immunological parameters that are measurable in the platform utilized in this study. IL-17 and IL-8 are factors associated with the recruitment and activation of neutrophils, which may contribute in the development of corneal haze [13,14].

Previous reports have studied the inflammatory processes in animal models of corneal transplant and wound healing, however studies in humans have been limited. We hypothesize that the coordinated, stepwise production of these factors critical for successful corneal repair will be reflected in tear fluid profiles of transplant cases. We have thus screened profiles of soluble factors in the tear fluid of the transplant and non-transplant eyes over the course of post-transplant wound healing. Tear fluid profiling has promise to monitor the process of transplant recovery and to guide customized treatment against complications.

Through these analyses, we have identified (1) the kinetics of 51 bilateral tear proteins after endothelial keratoplasty, (2) a set of tear fluid proteins that were associated with preexistent corneal haze, and (3) those that have the potential to predict delayed clearance of corneal haze and visual acuity after endothelial keratoplasty.

## 2. Experimental Section

### 2.1. Human Subjects, Patient Characteristics, and Clinical Specimens

Twenty-one patients who underwent DSAEK were recruited at the Singapore National Eye Centre from 2013 to 2015 (Table 1). The average age was 69 years (30–91). The transplant patients were distinguished by underlying cause of corneal opacity: two cases had Fuchs’ endothelial dystrophy (FED), and 19 cases had bullous keratopathy (BK). More than half of the BK patients had past histories of glaucoma treatment or cataract surgery. The two FED patients received DSAEK in their contralateral eyes more than three years earlier. DSAEK was performed following the standard techniques [15]. The study was conducted in accordance with the Declaration of Helsinki and was approved by the Singhealth Institutional Review Board. Written informed consent was received from all participants. We collected tear fluid from patients before the surgery (D0), as well as one day (D1), one week (W1), and one month (M1) after the surgery. Tear fluid from both the transplant and non-transplant eyes was collected at the same time. Tear fluid collection used Schirmer strip filter paper as described previously [16]. Clinical records were collected with the following information: status of corneal epithelial defect, conjunctival congestion, conditions of corneal stromal haze [Fantes grading; scored from 0 to 4; none (0), minimal (1), mild (2), moderate (3), and severe (4)], inflammation status in the anterior chamber, and LogMAR visual acuity. After DSAEK, all patients received topical corticosteroids (0.1% Dexamethasone, Minims Dexamethasone sodium phosphate 0.1%) in their transplant eyes every three hours for one month. Graft rejection was observed in two patients during the follow up after surgery (average follow up period: 22 months, range 3–39 months). As a healthy control group, tear samples were collected from four age-matched healthy individuals without prior history of ocular surface diseases and surgery.

### 2.2. Tear Protein Extraction

Tear-fluid volume was estimated from the length of the wet portion of the Schirmer strip as described previously [16]. The Schirmer strips were soaked in PBS containing 0.5 M NaCl, 0.3% Tween 20, 0.5% bovine serum albumin, and proteinase inhibitor at 4 °C overnight before the supernatant was collected. Tear fluid samples of less than 1 μL were excluded from the protein analysis.

### 2.3. Multiplex Analysis of Cytokines and Chemokines in Tear Fluid

Fifty-one cytokines, chemokines, and growth factors present in the tear fluid were analyzed using a magnetic-bead-based multiplex assay (Bio-Plex^TM^ 200 System; Bio-Rad Laboratories; Hercules, CA, USA). The analytes were IL-1a, IL-2Ra, IL-3, IL-12 (p40), IL-16, IL-18, CTAK, GRO-a, HGF, IFN-a2, LIF, MCP-1, M-CSF, MIF, MIG, NGF, SCF, SCGF-b, SDF-1a, TNF-b, TRAIL, IL-1b, IL-1Ra, IL-2, IL-4, IL-5, IL-6, IL-7, IL-8, IL-9, IL-10, IL-12p70, IL-13, IL-15, IL-17, Eotaxin, FGF basic, G-CSF, GM-CSF, IFN-g, IP-10, MCP-1, MIP1-a, PDGF-bb, MIP-1b, RANTES, TNF-a, VEGF, TGF-b1, TGF-b2, and TGF-b3 (Table 2). The calibration of the assay system and measurements was performed by following the manufacturer’s instructions.

### 2.4. Principal Component Analysis of Tear Protein Expression Kinetics

Principal component analysis (PCA) of tear-soluble factors was conducted in R version 3.2.1. The mean value of factor *i* at time point *t*, $\mu_{t}^{i}$ was firstly calculated from all participants. Thereafter standardization (or Z-score normalization) of $\mu_{t}^{i}$ was conducted for each factor among different time points using the equation $Y_{t}^{i}=\frac{\mu_{t}^{i}-\;\mu^{i}}{sd},$ where $\mu_{t}^{i}$ refers to the measured value of tear-soluble factors *i* at time point *t*, *μ^i^* and sd refers to the mean and standard deviation of factor *i* across all the time points, respectively, and $Y_{t}^{i}$ is the standardization factor *i* at the time *t* value in the patient value. PCA was conducted using the PCA function in *FactoMineR* package. Clustering of all standardized values was conducted based on the silhouette coefficients, which is a measure of similarity of an object for its own cluster compared to other clusters [17]. The coefficient ranged from −1 to 1 and our optimal cluster size was achieved through maximizing the silhouette coefficients (closer to 1). The function *pamk* from package *fpc* was applied to evaluate the size. Optimal cluster size was obtained through partitioning all the points $Y_{t}^{i}$ around medoids to estimate the number of clusters using the *pamk* function.

### 2.5. Measurement of Tear Substance P

Tear substance P was measured via competitive enzyme immunoassay (R&D Systems, Minneapolis, MN, USA) according to the manufacturer’s instruction. Briefly, 50 μL of diluted tear samples were added to each well and incubated with primary antibody and horseradish peroxidase-labeled substance P for 3 h at room temperature. The excess conjugate and unbound samples were removed by washing, and substrate solution was added to the wells to determine activity of the bound enzyme. Color development was then stopped, and the absorbance was measured at 450 nm. Substance P concentration was normalized against the total protein of each sample.

### 2.6. Statistical Analysis

Data were statistically analyzed using Prism software (GraphPad Software; San Diego, CA, USA). Mann–Whitney tests were used for comparisons between two groups. Using a two-tailed test, *p*-values of <0.05 were considered significant. Correlation between cytokine expression and LogMAR visual acuity was calculated using Pearson correlation coefficients.

## 3. Results

### 3.1. Dynamic Changes in Tear Fluid Cytokines in Both Eyes after Endothelial Keratoplasty

**Figure 1:**
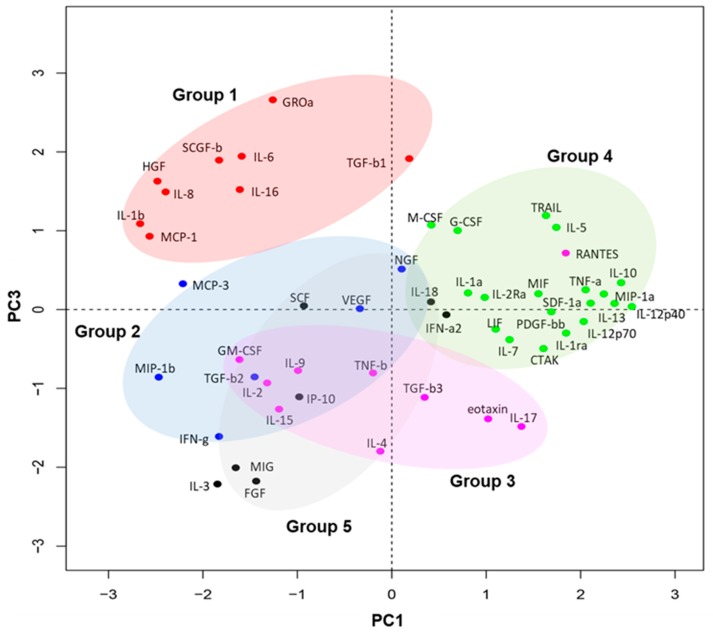
Five groups of soluble factors as classified by the kinetics in tear protein expression following EK. To enable visualization of groups of factors that displayed similar profiles of change over the course of transplantation, we implemented dimensionality reduction and a clustering algorithm. The mean of each of the 51 tear-soluble factors was calculated and normalized amongst the 21 patients using Z-score normalization for each time point (D0, D1, W1, M1). Principal component analysis was then conducted on the standardized time-point factors. The most informative clustering was visualized when PC1 and PC3 components were mapped onto a biplot (cumulative variance = 49.72%), and clustering was conducted with partitioning around medoids with the number of clusters estimated by optimum average silhouette width. The five clusters are color-coded (Details in the Experimental Section).

Tear fluid profiles displayed significant, complex changes over the course of recovery after transplant. To enable understanding of the alterations in tear fluid protein levels, we first classified groups of tear fluid proteins that displayed similar kinetics using principal component analysis (PCA), based on similarity in patterns of changes in expression levels over the four time points in the transplant and contralateral eyes, using values normalized between the 21 cases (details in the Experimental Section). Through this analysis, the 51 factors were classified into five groups (Figure 1). Of note, group 1 (Figure 1, cluster in red) was most distinct from the other four clusters, and comprised nine proteins which are known for their roles in innate immunity (IL-8, IL-6, MCP-1, GROa) and corneal wound healing (TGF-b1, IL-1b, HGF).

**Figure 2:**
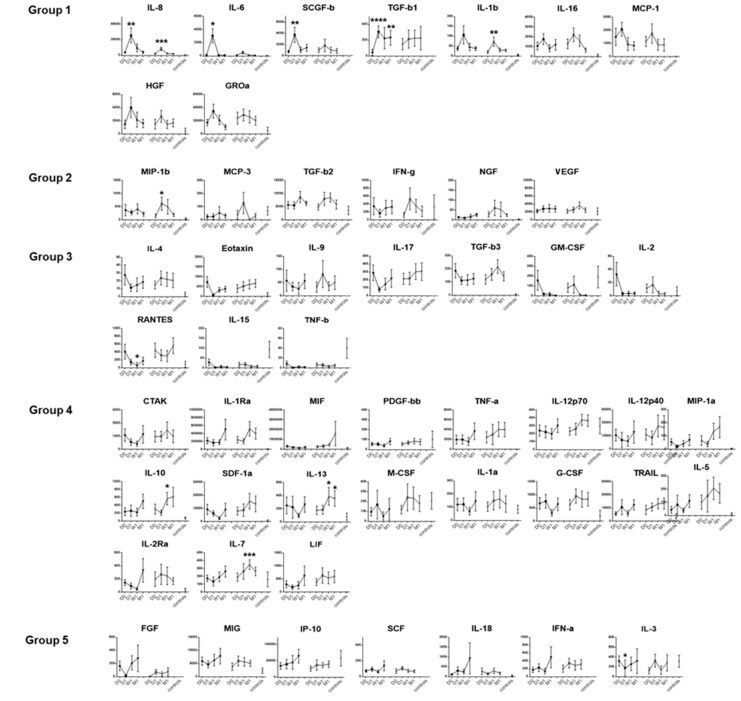
Dynamic change in tear fluid cytokines in bilateral eyes after EK. The mean ± SEM of tear protein levels at Day 0, Day 1, Week 1, and Month 1 time points are shown pairwise for the transplant and contralateral eyes in each plot for the 51 factors screened. Data shown are in pg/mL. The mean ± SEM are shown. * *p* < 0.05, ** *p* < 0.02, *** *p* < 0.01, **** *p* < 0.001.

**Figure 3:**
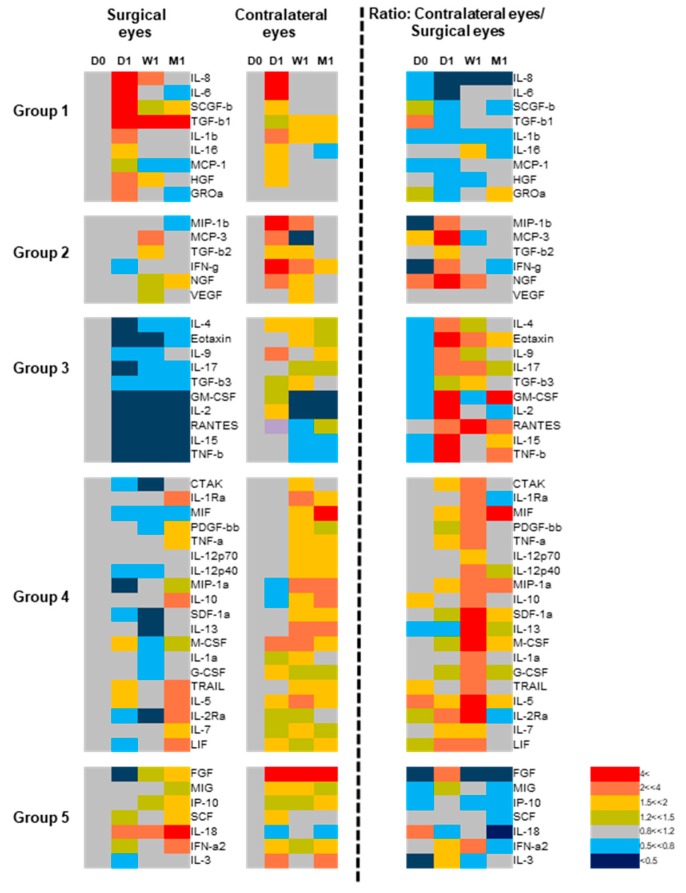
Five distinct kinetics of tear fluid cytokines in bilateral eyes after EK. Left and middle heat maps; surgical and contralateral eye tear protein expression kinetics at D1, W1, and M1 are shown in relative to the pre-transplant D0 baseline level. Panel on the right: The ratio of tear protein levels in the contralateral eyes against surgical eyes are shown for each time point. The grouping of the 51 proteins into five are based on the PCA analyses described in Figure 1. Transplant eyes: D0: *n* = 19, D1: *n* = 18, W1: *n* = 17, M1: *n* = 15. Contralateral eyes: D0: *n* = 19, D1: *n* = 17, W1: *n* = 17, M1: *n* = 15.

Next, we investigated how the levels of tear-fluid proteins changed over the four time points, individually by factor and by the surgical and the contralateral eye (Figure 2). To enable interpretation of the grouping of the 51 factors (Figure 1) and the various patterns of expression kinetic profiles (Figure 2), we used a heatmap representation of the dataset (Figure 3). Through these analyses, we found that the levels of proteins in Groups 1 and 2 tended to increase in both eyes. Group 1 proteins were those that displayed the peak primarily in the surgical eye, and the same pattern held to a lesser extent in the contralateral eye, peaking at one day after surgery. In contrast, the kinetics profile for the Group 2 proteins (MIP-1b, MCP-3, TGF-b2, IFN-g, NGF, and VEGF) displayed an increase in the contralateral eyes (rather than the surgical eye) immediately after surgery. The contrasting effects on different eyes become clear when the ratio of contralateral eye/surgical eye levels are represented (column on the right in Figure 3). These patterns inferred factors directly affected by the surgery in the transplant eye (Group 1) and the contralateral eye (Group 2). Groups 3 and 4 proteins were those that tended to display disparity in profile kinetics between the transplant and contralateral eyes, where prominent and mild decreases (Groups 3 and 4, respectively) were detected in the levels following surgery in the transplanted eyes. Conversely, protein levels tended to increase in the contralateral eyes. The soluble factors in Groups 3 and 4 comprised those mainly involved in immunological responses relating to adaptive immunity and included the majority of the factors analyzed in the study. These patterns inferred suppression of the adaptive immune system in the transplant eye, likely contributed by the post-transplant treatment protocol. However, a difference in the kinetics becomes apparent when the ratio of contralateral eye/surgical eye levels are represented (column on the right in Figure 3), where Group 4 proteins display a delayed effect as compared to Group 3 proteins. Group 5 protein levels tended to remain unchanged in the transplant eye until the last time point, whereas the levels in the contralateral eye tended to increase immediately after transplant.

### 3.2. The Effect of Corticosteroids on Tear Protein Expression

**Figure 4:**
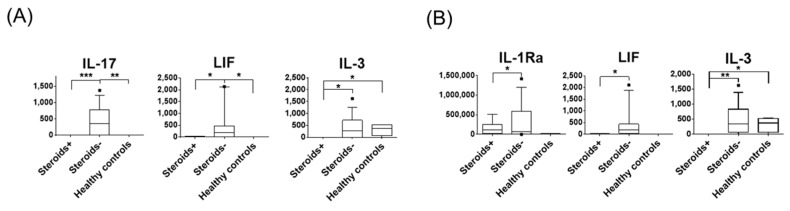
Effects of corticosteroids on baseline cytokine expression. Baseline tear protein expression was compared between transplant eyes (**A**) and contralateral eyes (**B**) with topical corticosteroids, without corticosteroids, and in age-matched controls. Corticosteroids^+^: *n* = 6, corticosteroids^−^: *n* = 15, and age-matched controls: *n* = 4. The proteins presenting significant differences between the corticosteroids^+^ and corticosteroids^−^ groups are shown. The median, 10%, 25%, 75%, and 90% percentiles are shown. pg/mL. * *p* < 0.05, ** *p* < 0.02, *** *p* < 0.01.

Corticosteroids are used as immunosuppressants in various inflammatory conditions, however, which cytokines are actually suppressed by corticosteroids is not well understood. Among the 21 eyes that underwent corneal transplantation, six eyes were treated with topical corticosteroids before the surgery. Therefore, we compared the tear protein expression between the eyes with and without pre-transplant corticosteroid treatments. Notably, the average corneal stromal haze scores were not significantly different between the two groups (1.5 and 2.4 in the eyes with and without corticosteroids, respectively). We found that the baseline levels (D0) of IL-17, IL-3, and LIF were significantly lower in the eyes as compared to those without corticosteroid treatment (Figure 4A). The eyes without corticosteroid treatment showed increased levels of IL-17 and LIF compared to those in healthy controls. Notably IL-17, IL-3, and LIF levels decreased after keratoplasty in the surgery eyes that were treated with topical corticosteroids, as shown in Figure 2, suggesting that corticosteroids can lower tear fluid cytokine levels, and IL-17, IL-3, and LIF are those clearly affected among the 51 soluble factors. The levels of IL-3 and LIF were also lower in the contralateral eyes with pre-transplant topical corticosteroid treatments (Figure 4B).

### 3.3. Bilateral Increase in Tear Cytokines in Patients with Unilateral Corneal Haze

**Figure 5:**
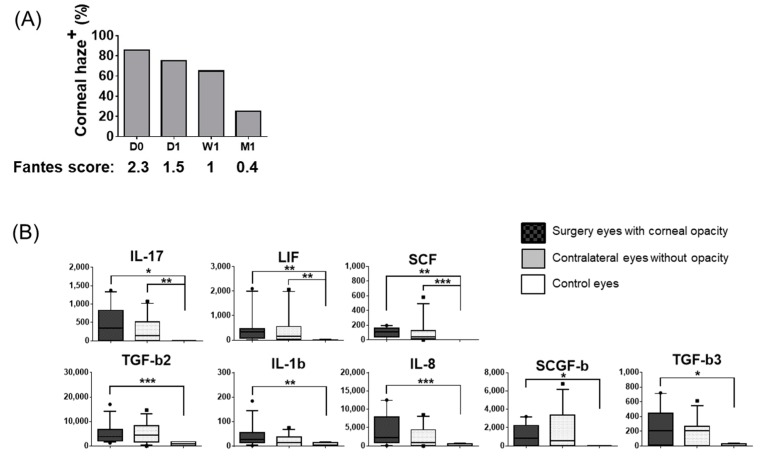
Tear fluid cytokines increased in bilateral eyes with unilateral corneal haze. (**A**) Frequencies and average scores of corneal haze in the transplant eyes at each time point are shown. *n* = 21. (**B**) Baseline protein expression levels were compared between transplant eyes with corneal haze, non-transplant eyes without corneal haze, and age-matched controls. Patients receiving corticosteroid treatment before surgery were excluded. Corneal haze^+^ transplant eyes: *n* = 11, corneal haze^−^ non-transplant eyes: *n* = 11, age-matched controls: *n* = 4. The median, 10%, 25%, 75%, 90% percentiles are shown. pg/mL. * *p* < 0.05, ** *p* < 0.02, *** *p* < 0.01.

We noticed the presence of corneal haze in 18 of the 21 (85.7%) eyes prior to DSAEK (Figure 5A). An important question thus raised was whether tear cytokine levels associated with preexisting corneal stromal haze. To this end, we compared the baseline tear fluid cytokine levels between the eyes with stromal haze that underwent surgery versus the contralateral eyes without corneal haze. Patients receiving corticosteroid treatment before surgery were excluded. The result suggested no significant association among the 51 tear proteins in this context. However, when we compared the tear fluid protein levels of patients with preexisting corneal haze, versus those of healthy controls, we found that the levels of eight tear proteins (IL-17, LIF, SCF, TGF-b2, IL-1b, IL-8, SCGF-b, and TGF-b3) were significantly higher in the transplant eyes compared to the healthy controls (Figure 5B). Three of the eight proteins belonged to Group 1.

Notably, all of these factors were bilaterally elevated, among which IL-17, LIF, and SCF showed significant increases in the contralateral eyes compared to those in healthy controls. Thus bilateral tear fluid cytokine changes occurred in patients with unilateral corneal haze. Importantly, these eight tear fluid proteins were among the factors with increased levels in the contralateral eyes after EK (Figure 2).

### 3.4. Baseline and One Week-Post Transplant Cytokine Levels Are Major Determinants of Corneal Haze at One Month after Endothelial Keratoplasty

Since the introduction of endothelial keratoplasty, the fast recovery of corneal transparency has become the prime concern after surgery [18]. Factors associated with corneal graft, as well as clinical phenotype, are known risk factors for inferior visual recovery after EK, but no molecular factors have been reported [18,19]. Therefore, we asked whether tear fluid protein profiles could be used to predict delayed recovery from corneal haze following surgery. Corneal haze disappeared completely in the majority of the eyes one month after surgery, however, it remained in five eyes (25%) (Figure 5A). Minimal haze was observed in four of the five eyes (the average score 1.6) and no corneal haze was observed at the final clinical examination.

**Figure 6:**
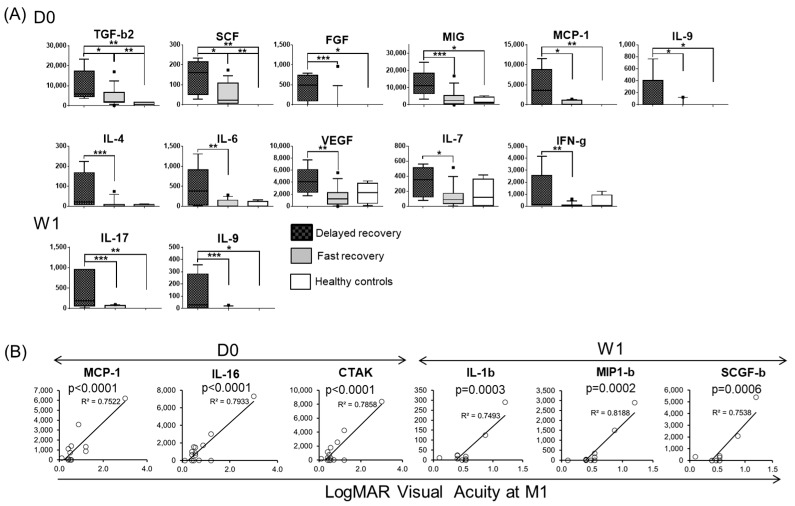
Tear protein expression at Day 0 and Week 1 associated with recovery after EK. (**A**) The expression levels of proteins significantly associated with rapid recovery of corneal haze, measured one month post-transplant, are shown. The delayed recovery group (Corneal haze^+^ at Month 1 (M1); *n* = 5), and the fast recovery group (non-corneal haze at M1; *n* = 15), age-matched controls: *n* = 4. Data are shown in pg/mL. The median, 10%, 25%, 75, 90% percentiles are shown. * *p* < 0.05, ** *p* < 0.02, *** *p* < 0.01. (**B**) The correlation between tear protein expression and LogMAR visual acuity measured one month post-transplant. Proteins significantly associated with visual acuity are shown. *n* = 15.

Indeed, we found that particular baseline tear fluid protein profiles were associated with recovery from corneal haze within one month after surgery. At each time point, we compared tear cytokine levels between two groups based on their recovery status following EK; a fast recovery group (one with no corneal haze) and a delayed recovery group (the other presenting corneal haze) at one month post-transplantation (Figure 6A). We found that the levels of eleven tear fluid proteins were significantly higher in the delayed recovery group as compared to the fast recovery group (TGF-b2, SCF, FGF, MIG, MCP-1, IL-9, IL-4, IL-6, VEGF, IL-7, and IFN-g) at the pre-transplant baseline time point. Furthermore, two tear cytokines (IL-17 and IL-9) at one week after surgery were significantly higher in the delayed recovery group compared to the fast recovery group (Figure 6A). Tear IL-9 was elevated in the delayed recovery group both in the baseline and after one week but showed no difference at one month. Most of these proteins are factors known to contribute to corneal wound healing or fibrotic wound healing in various organs [20,21].

There were no significant differences in tear protein expression at one day/one month after surgery between the two groups. Furthermore, there was no significant association in protein levels between the transplant eyes with and without haze at any time points. No association was observed in delayed recovery from corneal haze with the degree of corneal epithelial defect, conjunctival congestion, and the inflammation status of the anterior chamber.

### 3.5. Tear Protein at Baseline and One Week after DSAEK Are Associated with Visual Acuity at One Month Post-Surgery

We then sought to determine whether tear fluid cytokine profiles were associated with differences in visual acuity after EK. We found that the higher levels of (1) MCP-1, IL-16, and CTAK at baseline, and (2) IL-1b, MIP-1b, and SCGF-b at one week post-transplant displayed strong associations with lower visual acuity at one month post-surgery (Figure 6B). Four of the six proteins belonged to Group 1. This association was observed only in those cases without significant retinal/optic neuronal damage; no significant association was observed between the tear fluid cytokines and visual acuity when the transplant cohort was analyzed as a whole. This result infers that changes in tear cytokines have the potential to be used to prognosticate visual acuity when corneal haze is the main factor impairing visual acuity.

Finally, we asked whether tear substance P expression was associated with tear inflammatory cytokine levels after surgery. Upon analyzing substance P expression in the tear fluid of eight patients, we found that substance P was present in tear fluid at a high concentration before surgery, however, there was no significant change after surgery (Supplementary Materials Figure S1).

## 4. Discussion

To our best knowledge, this is the first report studying the bilateral tear fluid protein levels of eyes over the course of wound healing after corneal endothelial keratoplasty. Substantial changes in tear protein expression were observed in the ipsilateral and contralateral eyes, and the bilateral tear fluid protein expression profiles were classified into five groups. The expression levels of innate pro-inflammatory cytokines and chemokines, including IL-1b, IL-6, IL-8, and MCP-1, were immediately elevated in both eyes post-surgery, and this expression was not suppressed by corticosteroids. By contrast, expression of all the studied cytokines associated with adaptive immunity such as IL-2, IL-4, and IL-17 decreased in the transplant eyes immediately after EK.

The results of the tear fluid protein profiles in the transplanted eyes agree with a previous report that studied tear protein kinetics after photorefractive keratectomy [22]. In this report of 29 proteins, only IL-6, IL-8, and MCP-1 were increased, and the other 26 protein levels decreased after surgery. By contrast, several studies using animal models of corneal transplantation reported significant increases in various pro-inflammatory cytokines, including IL-1a, TNF-a, RANTES, MIP-1a, MIP-1b, and MCP-1 [23,24]. Only MCP-1 among these six cytokines increased, and the other five cytokines showed a reduction after surgery in our human study. The differences in expression profiles between the human and animal studies are likely due to corticosteroid use in the clinic. The tear fluid protein expression returned to the baseline levels at one month. The exceptions were several immunoregulatory proteins such as TGF-b1 and IL-13, inferring the potential roles in maintaining immunotolerance after corneal transplantation.

Th1 and Th17 cytokines have been associated with corneal graft rejection [25]. Rejection was observed in only two cases during the follow up, implying that the observed tear cytokine kinetics represents the proper profiles seen in normal wound healing without rejection.

The clinically important and unique involvement of the immune system in corneal transplantation is the phenomenon behind corneal graft rejection, often triggered in the contralateral eye, when a previously transplanted cornea follows a new transplantation in the other eye. This study highlights the effects of the EK procedure upon the contralateral eye, as evidenced by an increase in pro-inflammatory cytokines. Increased cytokine expression in the contralateral cornea has been reported in animal models of penetrating keratoplasty (IL-1Ra, MCP-1, and CCL20) and chemical injury (IL-1b, TNF-a, and VEGF). A significant increase in tear fluid cytokines has been reported in the contralateral eye, in unilateral infectious keratitis cases in humans [26,27]. Compared to the ocular conditions reported above, our cohort received a substantially less invasive procedure, and topical corticosteroids were used after surgery in all cases. Nevertheless, changes in tear fluid cytokine levels were clearly detected in the contralateral eye following EK. Whether the observed changes in particular cytokine expression are unique to EK or are also observed in other less invasive surgeries, such as for cataracts, is not clear in this study and should be determined in future studies.

The mechanism of how injury/surgery to one eye affects the other eye is a longstanding question of biological and clinical importance. The corneal nerve-trigeminal route has recently been suggested as a mechanism transmitting inflammatory signaling from one eye to the contralateral eye in animal models [28,29]. Our study provides evidence that a broad spectrum of cytokines and chemokines is involved in this bilateral ocular immune response.

It is important to consider the effects of topical corticosteroid administration in the transplant eye after endothelial keratoplasty. The higher levels of Group 2–4 tear fluid cytokines detected in the contralateral eyes are likely the consequence of corticosteroid-mediated immune suppression in the transplant eye. It is worth highlighting that in one transplant case, topical corticosteroids were administered pre-transplant in the contralateral eye, and the tear fluid protein profiles and their kinetics were identical to those of other cases in the contralateral eye. This gives rise to the speculation that corticosteroids directly suppress the inflammatory response in the eyes exposed to direct injury/surgery, while the inflammation that is indirectly induced in the contralateral eyes is resistant to corticosteroids.

A second clinically relevant finding of this study is the correlation between unilateral corneal haze and bilaterally elevated levels of eight tear fluid pro-inflammatory cytokines, chemokines, and growth factors at baseline prior to surgery. These factors included fibrotic (TGF-b2), anti-fibrotic (LIF, TGF-b3), pro-inflammatory (IL-1b), recruitment/activation of neutrophils (IL-8, IL-17), and immune-regulatory (TGF-b2, LIF) proteins [10,14,30]. SCGF-b levels were also significantly increased after EK. SCGF-b is a stem cell growth factor, of which the roles in wound healing and corneal haze remain unknown. All eight proteins showed immediate elevation in the contralateral eyes after normal EK. We hypothesize that the contralateral ocular inflammation in the acute phase after corneal injury/surgery and in donors with chronic unilateral corneal haze are both mediated by the same mechanism involving these eight factors.

Previous animal studies have suggested that substance P, a neurotransmitter, is involved in the inflammation of the contralateral eyes after chemical injury and penetrating corneal transplantation [28,29,31]. However, we were not able to detect significant changes in the level of substance P in the tear fluid over the course of corneal healing. We speculate that in humans, other neurotransmitters may also play the role of transmitting inflammation between the eyes. Alternatively, tear fluid may not be a suitable sample for evaluating changes in substance P expression, which is released by the corneal trigeminal neurons [32].

Third, an unexpected finding is that tear cytokine profiles have the potential to predict delayed recovery from corneal haze and of visual acuity. Notably, this study infers that tear protein profiles at both the baseline and one week after EK are useful in determining the early visual prognosis after EK.

Other factors such as topical treatments other than corticosteroids and pseudophakia could affect the tear cytokine levels. This could be determined through more in-depth analysis comparing various subgroups in future studies with larger cohorts.

The difficulty in analyzing multiple parameters within a limited amount of tear fluid was overcome by implementing a multiplexed bead-based system that has been utilized successfully in studies of various conditions such as keratoconus, dry eye, and allergic conjunctivitis [16,33,34,35,36,37,38]. The methodology to collect tear fluid using the Schirmer strip has been widely used in past studies, however, caution should be taken, for example, to prevent reflexive tearing due to irritation and due to unknown effects of diluents.

## 5. Conclusions

In this study, we have exemplified the potential usefulness of tear cytokine profiles in corneal transplantation. The kinetics of cytokine expression in bilateral tear fluid after corneal transplantation provide the basis of the targets that immunosuppressive drugs should act upon. Profiles of tear proteins at baseline and one week post-surgery are valuable to predict the recovery from corneal haze and visual prognosis after surgery. Tear proteins associated with baseline corneal haze are potential targets when developing new therapeutics for corneal haze.

## Figures and Tables

**Table 1:** Summary of the endothelial keratoplasty (EK) cases in this study.

	Age	Gender	Indication	History of Corneal Transplantation		Presurgery Topical Treatment		IOL	History of Glaucoma Treatment	Neuroretinal Damage	Recovery	Rejection in This Study
					Rejection	Steroids	Others					
1	72	F	BK				Timolol	+	+	+	Delayed	
2	74	F	BK	PKP	+	+					Delayed	+
3	76	F	BK			+					Fast	
4	85	M	BK							+	NA	
5	91	F	BK						+		Fast	
6	81	F	FED	DSAEK (contralateral eye)							Fast	
7	58	M	BK	DSAEK		+		+		+	Fast	
8	75	F	BK								Fast	
9	65	F	FED	DSAEK (contralateral eye)				+			Fast	
10	72	M	BK	DSAEK	+		Timolol, Brimonidine		+		Delayed	
11	50	M	BK				Timolol		+		Fast	+
12	73	M	BK					+			Delayed	
13	83	M	BK						+		Fast	
14	75	F	BK								Fast	
15	78	F	BK					+	+		Fast	
16	38	M	BK			+		+	+		Fast	
17	30	F	BK			+		+			Fast	
18	64	M	BK			+					Fast	
19	61	M	BK						+		Delayed	
20	63	F	BK							+	Fast	
21	87	F	BK	DSAEK						+	Fast	

FED: Fuchs’ endothelial dystrophy, BK: bullous keratopathy, PKP: Penetrating Keratoplasty, DSAEK: Descemet’s stripping automated endothelial keratoplasty, IOL: intraocular lens.

**Table 2:** Analyzed tear soluble factors.

Cytokines				Chemokines	Growth Factors
Wound Healing	Adaptive Immunity		Innate Immunity		
	Proinflammatory	Immunoregulatory	Proinflammatory		
IL-1a *	IL-2	IL-4	IL-6	Eotaxin	SCGF-b
IL-1b *	IL-2Ra	IL-5	IL-8	IP-10	SCF
IL-1Ra *	IL-9	IL-10	IL-12 p40	MCP-1	B-NGF
TGF-b1 *	IL-16	IL-13	IL-12 p70	MIP-1a	LIF
TGF-b2 *	IL-17	TRAIL	IL-15	MIP-1b	IL-3
TGF-b3 *		MIF	IL-18	RANTES	IL-7
G-CSF			IFN-γ	MCP-3	GM-CSF
M-CSF			IFN-a2	MIG	
VEGF *			TNF-a	CTAK	
FGF *			TNF-b	GRO-a	
PDGF-bb *				SDF-1a	
HGF ^#^					

* Corneal epithelial cell-derived factors, ^#^ Keratocyte/fibroblast-derived factors.

## Supplementary Materials

The following are available online at https://www.mdpi.com/2077-0383/9/1/63/s1, Figure S1: Substance P expression levels in the tear fluid before and after EK.
