# Supplementary material for: Kinetics of Tear Fluid Proteins after Endothelial Keratoplasty and Predictive Factors for Recovery from Corneal Haze
Source: J Clin Med. 2019 Dec 26;9(1):63. doi: 10.3390/jcm9010063 (PMC7019256; doi:10.3390/jcm9010063)
Supplement: Supplementary file 1 [file jcm-09-00063-s001.pdf]

Supplementary Materials

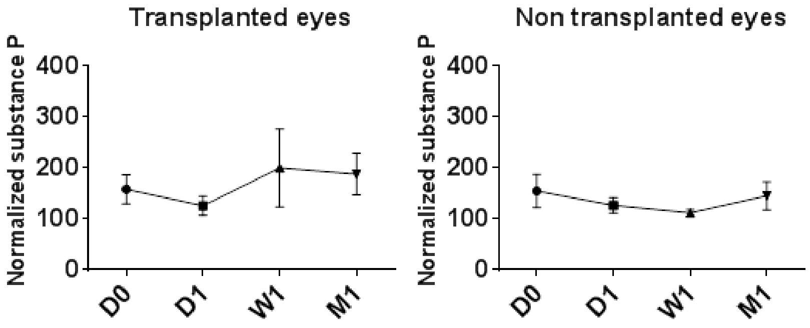

Figure S1. Substance P expression levels in the tear fluid before and after EK.

Mean values normalized with total protein expression are shown. n=8.
